# Comparison of the Mechanisms of Drug Resistance among HIV, Hepatitis B, and Hepatitis C

**DOI:** 10.3390/v2122696

**Published:** 2010-12-14

**Authors:** Severine Margeridon-Thermet, Robert W. Shafer

**Affiliations:** Departments of Medicine and Pathology, Stanford University, Stanford, CA 94305, USA; E-Mails: severine@stanford.edu (S.M.T.)

**Keywords:** HIV, HBV, HCV, antiviral therapy, drug resistance, evolution, quasispecies

## Abstract

Human immunodeficiency virus (HIV), hepatitis B virus (HBV), and hepatitis C virus (HCV) are the most prevalent deadly chronic viral diseases. HIV is treated by small molecule inhibitors. HBV is treated by immunomodulation and small molecule inhibitors. HCV is currently treated primarily by immunomodulation but many small molecules are in clinical development. Although HIV is a retrovirus, HBV is a double-stranded DNA virus, and HCV is a single-stranded RNA virus, antiviral drug resistance complicates the development of drugs and the successful treatment of each of these viruses. Although their replication cycles, therapeutic targets, and evolutionary mechanisms are different, the fundamental approaches to identifying and characterizing HIV, HBV, and HCV drug resistance are similar. This review describes the evolution of HIV, HBV, and HCV within individuals and populations and the genetic mechanisms associated with drug resistance to each of the antiviral drug classes used for their treatment.

## 1. Introduction

The human immunodeficiency virus (HIV), the hepatitis B virus (HBV), and the hepatitis C virus (HCV) each cause lifelong human infection and illness. HIV infects nearly 40 million persons and causes about two million deaths per year. HBV infects more than 400 million persons and causes nearly one million deaths per year. HCV infects nearly 200 million persons and is responsible for 50 to 75% of hepatocellular carcinomas in industrialized countries. Antiviral compounds targeting essential enzymes and other viral targets are either licensed or in advanced clinical development for each of these infections.

The development of drug resistance is the most compelling evidence that an antiviral drug acts by specifically inhibiting the virus rather than its cellular host. The genetic mechanisms of antiviral drug resistance are identified during the earliest stages of drug development by *in vitro* selection experiments and by *ex vivo* analysis of viruses obtained from individuals receiving antiviral therapy. This review describes the evolution of HIV, HBV, and HCV within individuals and populations and the genetic mechanisms associated with drug resistance to each of the antiviral drug classes (Table 1).

## 2. Viral Replication and Persistence

### 2.1. HIV

HIV enters CD4+ T lymphocytes in a three-step process: gp120 Env binds the CD4 receptor and induces a conformational change that enables it to also bind either the CCR5 or CXCR4 coreceptor. The formation of the gp120-CD4-coreceptor complex exposes the extended form of the transmembrane gp41 protein, which fuses the virus and host cell membranes. Following cell entry and viral disassembly, HIV RT converts two copies of single-stranded RNA into minus-strand DNA and then copies minus-strand DNA to create a DS DNA copy of the viral genome. Integrase (IN) catalyzes the cleavage of conserved dinucleotides from the 3’ ends of double-stranded HIV-1 DNA and remains bound to each of the 3’-ends as this circular pre-integration complex translocates to the nucleus. IN then catalyzes the strand transfer reaction, which leads to the integration of the HIV-1 genome into the host genome.

HIV integration is usually followed by viral transcription, translation, and maturation. The latter is characterized by the cleavage of Gag and Gag-Pol polypeptides by protease into the structural and enzymatic proteins of the newly created virus. However, in a certain proportion of infected cells, particularly in resting CD4+ T cells, HIV persists as an integrated proviral genome. Although many proviral DNA genomes are defective or irreversibly silenced by epigenetic mechanisms, many are also capable of reactivating particularly when the host cell undergoes immune stimulation. This proviral DNA reservoir decays slowly and is only minimally affected by antiretroviral therapy [1,2]. As a result, recurrent viremia and immunological decline ensue whenever therapy is discontinued regardless of the duration of previous virologic suppression.

### 2.2. HBV

In virions, the HBV genome is a relaxed circular DNA (RC-DNA) molecule that is only partially double stranded. After infection of a hepatocyte, RC-DNA is transported to the nucleus and converted by cellular enzymes into a covalently closed circular DNA molecule (cccDNA). cccDNA serves as the template for transcription and for pre-genomic RNA, which has two possible fates: (i) it can be encapsidated with HBV viral polymerase, serve as the template for minus-strand DNA and RC-DNA, and secreted extracellularly or (ii) it can be recycled back to the nucleus to amplify or replenish the cccDNA pool [3].

HBV cccDNA is highly stable. It can be eliminated by cell turnover, immune mechanisms, or possibly epigenetic silencing [4–7]. Nonetheless, most acutely infected adults clear their infection within six months coincident with the development of antibodies to the HBV envelope S protein (HBsAb seroconversion) and the disappearance of plasma HBsAg and viral DNA. In contrast, perinatally infected newborns, horizontally infected infants, and about 5 to 10% of immunocompetent adults develop persistent infection. Among individuals with persistent infection, spontaneous clearance is uncommon, occurring at a frequency of <1% per year [8].

### 2.3. HCV

HCV is a positive-sense, single-stranded enveloped virus with a genome of about 9.5 kb. The genome encodes a single large 9.0 kb open-reading frame flanked by conserved 5’- and 3’-untranslated regions. The 5’-untranslated region contains the internal ribosomal entry site (IRES), which is necessary for initiating translation. Viral replication occurs in a membrane-associated cytoplasmic replicase complex, consisting of the nonstructural proteins NS3, NS4A, NS4B, NS5A, and NS5B which directs the synthesis of a negative-strand copy of the genome. The resulting duplex RNA serves as a template for the synthesis of multiple copies of the positive-strand genome for protein production and packaging.

HCV persists in up to 70% of untreated infected persons [9–11]. There is a strong association of HCV clearance with genetic variation in the IL28B gene underscoring the importance of innate immunity in the host response to infection [12]. HCV’s life-long persistence in the majority of infected persons in the absence of treatment is a remarkable demonstration of its ability to evade the innate and adaptive immune system of its host. The absence of a stable intracellular reservoir (in contrast to HIV and HBV), however, makes viral eradication possible.

## 3. Virus Evolution in Individuals

Viral evolution within an infected host is determined by the number of viral replication cycles, the frequency of nucleotide incorporation errors, the potential for viral recombination, and host-mediated and antiviral selection pressures. Intra-host viral genetic diversity also depends on the time between initial infection and viral sampling and on whether the initial infection was clonal or composed of multiple heterogeneous clones.

Acute HIV infection has been shown to be clonal in the majority of infected patients and oligoclonal in the remaining patients [13,14]. Such data, however, are not generally available for HBV and HCV. Transmitted HIV drug resistance also occurs commonly in many parts of the world but is extremely rare for HBV and HCV.

Intra-host viral genetic diversity differs by genomic region with greater diversity occurring within genes encoding envelope proteins compared with structural or enzymatic proteins. Although synonymous mutation rates are usually greater than nonsynonymous mutation rates, synonymous mutations should not be assumed to be neutral because RNA viruses contain many functional genomic secondary structural elements and potentially structural constraints imposed by viral genome packaging [15,16].

HIV, HBV, and HCV are usually called quasispecies because they exist within individuals as highly heterogeneous virus populations that diversify during the course of infection. Although the extent of genomic diversity in these infections does not meet the original definition of a quasispecies, which requires an effectively infinite population size, population geneticists have nonetheless found quasispecies theory to be useful for finite viral populations with high mutation rates and have generally accepted the use of the term quasispecies when applied to HIV, HBV, and HCV infections [17,18].

### 3.1. HIV

HIV, like most lentiviruses, replicates throughout the course of infection. Plasma virus RNA levels range from 10^3^ to 10^6^ copies/mL in most untreated infected individuals. The plasma virus half-life is estimated to be about five hours and up to 10^10^ viruses are produced each day in untreated individuals [19].

Mutations occur at two stages of HIV replication: (i) when RT catalyzes the conversion of the two copies of single-stranded genomic RNA into DS DNA; and (ii) when host DNA-dependent RNA polymerase transcribes viral RNA from provirus. HIV mutation rates per replication cycle have been estimated using intracellular fidelity assays designed to detect either the inactivation of a reporter gene or the reversion of an inactivating mutation in a reporter gene [20]. Based on such studies, the HIV-1 nucleotide misincorporation rate has been estimated to be about 1 × 10^−5^, which is similar to that of other retroviruses [21,22]. However, not all nucleotide positions mutate at the same rate. Mutations occur at increased rates in homopolymeric regions [23].

Recombination is a feature shared among retroviruses. It occurs because RT switches between two co-packaged SS RNA genomes as it creates a single DNA copy. If the co-packaged SS RNA genomes were derived during infection of a single cell by viruses with different sequences, then recombination during the next cycle of replication produces mosaic viral sequences that may differ from the parental genomes at multiple nucleotide positions [24,25]. Recombination, therefore, has a high potential to shape HIV evolution [26], although its effect is limited by the requirement that different HIV variants infect the same cell and by the possibility that the recombinant progeny may not replicate as well as their non-recombinant precursors [27,28].

HIV is under constant selection pressure to avoid adaptive humoral and cellular host immune defenses, which results in a high frequency of mutation at HLA-compatible cytotoxic T lymphocyte (CTL) epitopes. In individuals infected with a single virus strain, genetic diversity usually increases progressively during the course of infection, by as much as 1% per year in the envelope gene [29]. In the absence of drug selection pressure, the rates of divergence are considerably lower in the enzymatic targets of therapy [30,31]. However, in patients receiving incompletely suppressive antiretroviral therapy, many mutations can develop within days to weeks or months.

APOBEC-mediated deamination of cytidines to uracil in negative-strand DNA molecules of retroviruses, retrotransposons, and hepatitis B is an innate host defense mechanism that results in a marked excess of G-to-A mutations (G→A hypermutation) in viral plus-strand DNA [32]. The antiretroviral effects of APOBEC3G (GG→AG) and APOBEC3F (GA→AA) are so significant that HIV is unable to replicate in the absence of Vif, a protein that neutralizes these enzymes. Although APOBEC-mediated G→A hypermutation of HIV usually results in nonviable viruses relegated to proviral DNA [33,34], the possibility that low-level APOBEC activity may have contributed to virus evolution is suggested by an increased frequency of nonsynonymous mutation at the dinucleotides typically targeted by APOBEC3G and APOBEC3F [35].

### 3.2. HBV

In the absence of therapy, plasma HBV DNA levels are often as high as 10^7^ to 10^9^ copies/mL and up to 10^11^ to 10^13^ virions per day may be produced within infected persons [36–38]. The half-life of HBV has been estimated to range from four hours for plasma viruses [39,40] to up to 24 hours for newly formed virions in the process of being released extracellularly [36,41].

HBV mutations accumulate within individuals at 10^−4^ to 10^−5^ substitutions per nucleotide per year [42–44]. On the basis of these data and comparisons with other hepadnaviruses, the mutation rate of HBV per round of replication is estimated to be about 10^−5^, a rate similar to that of HIV and other retroviruses [45,46]. Phylogenetic analysis of complete HBV genome sequences suggests that recombination has occurred at least several times during the virus’s evolutionary history [47]. However the mechanism by which HBV recombination occurs and the frequency with which it leads to the development of new variants within individuals are not known.

During acute infection, HBV faces selection pressure from the host’s innate and adaptive immune systems [48,49]. However, once infection is established, HBV often evolves to induce an immune tolerant state that may benefit the virus by allowing those infected perinatally to survive to adulthood and to transmit their infection to future generations.

Despite its high rate of mutation and replication, HBV’s evolution is constrained because nearly two-thirds of its genome encodes multiple proteins in overlapping reading frames [50,51]. Therefore, regardless of the rate at which mutations occur, the rate at which they become fixed is lower than that for HIV and HCV. Drug resistance, in particular, evolves much more slowly for HBV than for HIV or HCV because even in the presence of antiviral therapy, drug-susceptible viruses remain capable of producing intracellular virus. NRTI-resistant viruses however are more successful at replenishing the cccDNA pool and at infecting new hepatocytes—the two steps that require reverse transcription of pre-genomic RNA [52].

The mean number of nucleotide differences between plasma virus clones within antiviral-naïve infected persons is often less than 1% in the core and polymerase genes particularly during the immunotolerant stages of infection [53–55]. However, genetic diversity is higher during antiviral treatment failure [55–57] and possibly during acute infection and those stages of infection in which the virus is under immune selection pressure.

APOBEC mediated G→A hypermutation manifests differently in HBV than in HIV. First, it is not caused solely by APOBEC3G and APOBEC3F. Additional APOBEC enzymes—including APOBEC3C, which has no dinucleotide preference—appear to contribute to hypermutation [58]. Second, HBV does not appear to have a defense mechanism (such as Vif) against G→A hypermutation suggesting that APOBEC enzymes are not a critical threat to HBV replication. Third, hypermutated clones are detected at low levels (*i.e.*, 0.1% to 5.0%) in most clinical plasma samples [55,59]. HBV G→A hypermutation is important to recognize because certain drug-resistance mutations (A181T and M204I) are unlikely to be clinically significant if they occur in hypermutated, nonfunctional genomes [60].

### 3.3. HCV

HCV plasma levels typically range from 10^4.5^ to 10^6.5^ IU units/mL where one IU is about 1 to 5 RNA copies depending on the commercial assay used for quantification [61]. HCV has an estimated half-life of about three hours and, in the absence of antiviral therapy, up to 10^12^ virions are produced daily [62–65].

Like other RNA-dependent RNA polymerases (RdRp), HCV’s polymerase has a high error rate. Studies of virus evolution during point source outbreaks and over short time spans have shown that HCV accumulates about 1 × 10^−3^ nucleotide changes per site per year [66,67]. Based on these data and comparisons with related viruses, it has been estimated that 10^−4^ to 10^−5^ substitutions occur per nucleotide per round of replication [68], a mutation rate typical of non-retroviral RNA viruses.

Recombination occurs through a process of template switching during replication in many families of positive-strand RNA viruses. However, intra-host recombination has rarely been observed [69] and there have only been several documented inter-genotypic or inter-subtype recombinants [70–72]. The paucity of recombination may reflect the lesser fitness of recombinants compared with their parental strains due to mutational incompatibilities.

Adaptive humoral [73,74] and cellular [75,76] immunity create ongoing antiviral selection pressure throughout HCV infection. HCV also has multiple defense mechanisms against innate intracellular antiviral responses [77], but it is uncertain whether innate immunity influences HCV evolution within individuals. HCV’s ability to respond to external selection pressure is demonstrated by the rapidity with which it can develop resistance to small molecule inhibitors *in vitro* and *in vivo*.

HCV quasispecies become increasingly complex during the course of infection. On average, the genetic distance among genomes can range from 5 to 10% in NS5A or to greater than 10% in hypervariable regions of the envelope [78–81].

## 4. Virus Evolution in Populations

### 4.1. HIV

**Figure 1:**
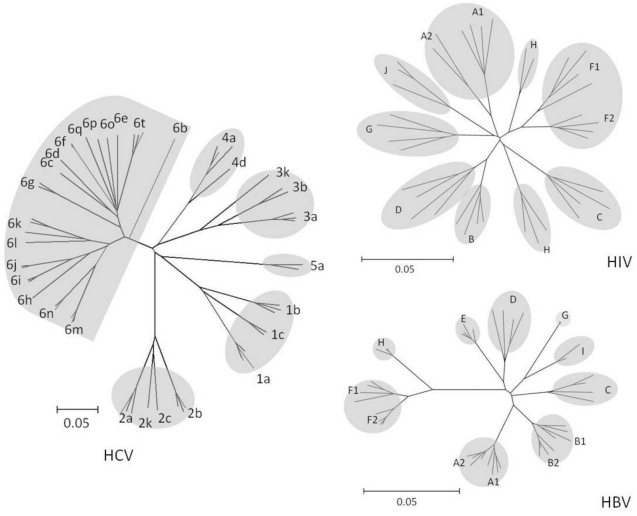
Phylogenetic Trees Created from HIV-1 Group M RT, HBV RT, and HCV Polymerase Sequences. The trees demonstrate the greater distances separating the HCV genotypes compared with those separating the HIV-1 group M subtypes and the HBV genotypes. Distances were calculated using the HKY85 substitution model with rate variation conforming to a gamma distribution. Trees were constructed using the neighbor-joining algorithm.

HIV-1 and HIV-2 are two of more than 15 primate lentivirus species that differ from one other by 40 to 60% of their amino acids. HIV-1 groups M and N represent cross-species transmissions from chimpanzees, whereas groups O and P represent cross-species transmission from chimpanzees or gorillas. HIV-1 group M is responsible for the worldwide HIV-1 pandemic; HIV-1 groups O, N, and P are extremely rare. Group M viruses began spreading among humans about 100 years ago and gave rise to multiple subtypes and well-characterized inter-subtype recombinants [82,83]. HIV-1 subtypes differ from each other by about 10 to 30% of their nucleotides throughout their genome. However, within the enzymatic targets of therapy, the inter-subtype diversity averages 10 to 12% at the nucleotide level and 5 to 6% at the amino acid level (Figure 1).

The NRTIs, INIs, and—to a lesser extent—the PIs are active against HIV-2 strains *in vitro* and *in vivo* and are likely to be active against the rare non-M HIV-1 groups. In contrast, the NNRTIs and the fusion inhibitor enfuvirtide appear to be consistently active only against group M viruses. CCR5 inhibitors should theoretically be active against all HIV-1 strains that must bind the CCR5 receptor. There do not appear to be any consistent differences among group M subtypes in their susceptibility to the six antiretroviral drug classes [84]. However, there are several differences among the subtypes in their propensity to developing specific drug resistance mutations [85–95].

### 4.2. HBV

HBV infects humans and non-human primates. There are at least eight HBV genotypes, which differ from one other by approximately 8 to 10% of their nucleotides. However, because primate HBV sequences are very similar to non-primate HBV sequences, it is possible that multiple cross-species transmission events occurred and that current HBV strains in humans do not have a single common human virus ancestor (Figure 1).

With the exception of genotype G viruses, which contain a 36-bp insertion in the core gene and two pre-core stop codons and which usually occurs in combination with genotype A viruses [96], there are no proven biological differences among the genotypes. Although several studies have suggested that the HBV genotype may influence disease progression and response to Interferon therapy, few data suggests that genotype influences viral response to NRTI therapy [97–101].

### 4.3. HCV

There are six major genotypes that differ in their nucleotide sequence by 30% to 35%. Within genotypes, subtypes differ by 20% to 25% [102,103]. Although HCV shares the same basic genomic organization as other flaviviruses, they are only distantly related and the origin of HCV is uncertain (Figure 1). Although there appear to be no differences in clinical severity among the various genotypes, there are major differences in the response to IFN-based therapy [104–106]. Small molecule inhibitors have been targeted towards genotype 1 because this genotype is the most difficult to treat with IFN and Ribavirin and is the most prevalent genotype in the U.S. and Europe.

## 5. HIV Drug Resistance

Twenty-four antiretroviral drugs belonging to six mechanistic classes have been licensed for HIV-1 treatment: Seven nucleoside and one nucleotide RT inhibitors (NRTIs), nine protease inhibitors (PIs), four non-nucleoside RT inhibitors (NNRTIs), one fusion inhibitor, one IN inhibitor (INI), and one CCR5 inhibitor. In previously untreated individuals infected with drug susceptible HIV-1, combinations of three drugs from two drug classes leads to prolonged virus suppression and, in most patients, immune reconstitution. Once complete HIV-1 suppression is achieved, it usually persists indefinitely as long as therapy is not interrupted [107].

HIV-1 drug resistance may be acquired or transmitted. It is acquired in patients in whom ongoing virus replication occurs in the presence of suboptimal antiviral therapy. Although suboptimal antiviral therapy was once a consequence of an insufficient number of active drugs, it now usually results from treatment interruptions or incomplete adherence. Transmitted drug resistance accounts for about 15% of new infections in the U.S. [108], 10% in Europe [109], 5% in South and Central America, and less than 5% in most parts of Sub-Saharan Africa and South and Southeast Asia [110,111].

### 5.1. Nucleoside/Nucleotide RT Inhibitors (NRTIs)

The NRTIs are prodrugs that must be triphosphorylated—or in the case of the nucleotide Tenofovir (TDF) diphosphorylated—to their active form. This dependence on intracellular phosphorylation complicates the *in vitro* assessment of NRTI activity because phosphorylation occurs at different rates in different cell types and leads to discordances between *in vitro* and *in vivo* NRTI potency. Specifically, differences in the intracellular dNTP pools between the highly activated lymphocytes used for susceptibility testing and the wider variety of cells that are infected *in vivo* explain why NRTIs differ in their dynamic susceptibility ranges and in their clinically significant levels of *in vitro* resistance [112,113]. Clinical isolates from persons failing NRTI therapy may have several-hundred-fold reductions in susceptibility to Zidovudine (AZT), Lamivudine (3TC), and Emtricitabine (FTC), but will rarely have more than five-fold reductions in susceptibility to Didanosine (ddI), Stavudine (d4T), and TDF. However, even slight reductions in *in vitro* susceptibility to this second category of drugs are clinically significant [114].

There are two biochemical mechanisms of NRTI resistance that are caused predominantly by mutations in the N-terminal polymerase-coding region of HIV-1 RT. One mechanism is mediated by discriminatory mutations that reduce the affinity of RT for an NRTI, preventing its addition to the DNA chain [115]. Another mechanism is mediated by ‘primer-unblocking’ mutations that favor the hydrolytic removal of an NRTI that has been incorporated into the HIV-1 primer chain [112,116]. Because they are selected by the thymidine analog inhibitors AZT and d4T, primer-unblocking mutations are also referred to as thymidine analog mutations or ‘TAMs’.

All recommended first-line treatment regimens include one of the two cytosine analogues—3TC and FTC. Although highly potent, each has a low genetic barrier to resistance. A single mutation, M184V, confers a greater than 200-fold decrease in susceptibility to these drugs. Although M184V limits the effectiveness of 3TC and FTC for salvage therapy, both of these drugs retain some benefit even in the presence of this mutation—possibly as a result of the decreased replication capacity of viruses with M184V or of the fact that M184V increases HIV-1 susceptibility to AZT, d4T, and TDF, drugs that have frequently been used in combination with 3TC and FTC.

The most common TAMs include M41L, D67N, K70R, L210W, T215Y/F, and K219Q/E. A subset of these mutations—M41L, L210W, and T215Y—is particularly important for causing cross-resistance to ddI, Abacavir (ABC), and TDF [117–119]. In patients receiving regimens without thymidine analogs, K65R and L74V have replaced the TAMs as the mutations that occur most commonly in combination with M184V. K65R causes low-level resistance to d4T, intermediate resistance to 3TC and FTC, and high-level resistance to ABC, ddI, and TDF; however, it increases susceptibility to AZT [120,121].

T69SSS and Q151M are multi-NRTI resistance mutations. T69SSS is a double amino insertion at HIV-1 RT position 69. It nearly always occurs with multiple TAMs, where it causes intermediate resistance to 3TC and FTC and high-level resistance to the remaining NRTIs [122,123]. Q151M usually occurs in combination with several otherwise uncommon mutations (A62V, V75I, F77L, and F116Y). It causes intermediate resistance to TDF, 3TC, and FTC, and high-level resistance to the remaining NRTIs [124,125].

Many additional accessory NRTI-resistance mutations have been described, including mutations in the C-terminal regions of HIV-1 RT [126–128]. Most of these C-terminal mutations appear to facilitate primer unblocking by slowing primer/template translocation or RNAseH activity [129,130]. A detailed review of the role of C-terminal mutations in HIV-1 RT drug resistance is also included in this issue [131].

### 5.2. Nonnucleoside RT Inhibitor Resistance (NNRTIs)

The NNRTIs inhibit HIV-1 RT allosterically by binding to a hydrophobic pocket close to the enzyme’s active site. This binding pocket is less well conserved than the enzyme’s active dNTP-binding site. As a result, group M viruses have greater inter-isolate variability in their susceptibility to NNRTIs than to NRTIs [132]. Three NNRTIs are commonly used: Nevirapine (NVP), Efavirenz (EFV), and Etravirine (ETR).

Many single mutations in the NNRTI-binding pocket confer high-level NVP resistance; several also confer high-level EFV resistance (Table 2). Resistance emerges rapidly when NNRTIs are administered as monotherapy, or in the presence of incomplete virus suppression, which suggests that NNRTI resistance is caused by the selection of rare pre-existing populations of mutant viruses within an individual. The administration of a single dose of NPV to prevent mother-to-child HIV transmission routinely selects for NNRTI-resistant mutants that are detectable by standard sequencing for two months or longer [133,134].

A minimum of two mutations is required to cause high-level ETR resistance [135,136]. ETR’s increased genetic barrier to resistance is a result of its ability to adopt multiple biding modes within the NNRTI-binding pocket [137]. The NRTIs and NNRTIs are often synergistic. Several NNRTI-resistance mutations increase susceptibility to certain NRTIs [138] and several NRTI-resistance mutations increase NNRTI susceptibility [139,140].

### 5.3. Protease Inhibitors (PIs)

More than 80 non-polymorphic PI-selected mutations have been reported [127]. Most of these contribute to decreased *in vitro* susceptibility to one or more PIs [141,142]. The mutations with the greatest impact on susceptibility—D30N, V32I, G48V, I50V/L, V82A/T/L/F/S, and I84V/A—occur in the substrate cleft [142] reducing the binding affinity between the PI and the protease. However, several mutations in the enzyme flap, such as I54M/L, and in the enzyme core, such as L76V, and N88S, can also markedly decrease PI susceptibility (Table 2). Mutations elsewhere in the enzyme either compensate for the decreased kinetics of enzymes with active site mutations; cause resistance by altering enzyme catalysis, dimer stability, and inhibitor binding kinetics; or by re-shaping the active site through long-range structural perturbations [143,144]. Mutations at several protease cleavage sites are also selected during PI treatment, improving the kinetics of protease enzymes with PI-resistance mutations [145–148].

Ritonavir-boosted PIs, particularly lopinavir/r and darunavir/r have the highest genetic barrier to resistance among all antiretrovirals. Multiple mutations are required to compromise their antiretroviral activity [149–153].

### 5.4. Integrase Inhibitors (INIs)

Although IN catalyzes both the 3’-processing and strand-transfer reactions, only those compounds that specifically inhibit strand transfer are effective INIs [154,155]. The FDA-licensed INI raltegravir and two additional INIs in advanced clinical development—elvitegravir and S/GSK1349572—bind the essential divalent metal cations Mg^++^ or Mn^++^ and a hydrophobic region within a cavity formed by IN and the 3’ HIV-1 DNA ends [156,157].

Mutations at nine positions (T66I/A/K, E92Q/V, F121Y, Y143C/R, P145S, Q146P, S147G, Q148H/R/Q, and N155H/S) are selected by raltegravir or elvitegravir and reduce susceptibility to either one or both of these drugs by more than five-fold [158–163]. A large number of secondary compensatory mutations have also been described. The most important of these are G140S/A/C and E138K/A, which increase the fitness of viruses with Q148H/R/K and lead to high-level resistance to all INIs, and T97A, which causes high-level resistance to raltegravir in the presence of Y143C/R [163–166].

### 5.5. Fusion Inhibitors

Enfuvirtide is a synthetic peptide that inhibits fusion by binding to gp41’s HR1 region and preventing it from folding back and binding to its HR2 region [167]. Enfuvirtide-resistant isolates contain either single or double mutations between positions 36 and 45 of gp41 HR1 [168,169]. Single mutants typically decrease enfuvirtide susceptibility about 10-fold whereas double mutations decrease susceptibility about 100-fold. Despite being one of the most potent antiretroviral drugs, the genetic barrier to enfuvirtide resistance is low and virological rebound emerges rapidly if Enfuvirtide is not administered with a sufficient number of other active inhibitors [170].

### 5.6. CCR5 Inhibitors

Maraviroc allosterically inhibits the binding of HIV-1 gp120 to the seven-transmembrane G protein-coupled CCR5 receptor [171]. CCR5 inhibitor resistance develops during *in vitro* passage experiments via gp120 mutations that enable HIV-1 to bind to the CCR5—CCR5-inhibitor complex [172]. Resistance via this mechanism, however, does not occur rapidly nor does it occur by a consistent pattern of gp120 mutations. In patients receiving CCR5 inhibitors, the most common mechanism of virological failure is the expansion of pre-existing CXCR4 tropic viruses that are intrinsically insensitive to CCR5 inhibitors [173]. Less commonly, virological failure emerges via mutations that allow the virus to bind to the CCR5—CCR5-inhibitor complex [174–176].

## 6. HBV Drug Resistance

There are two forms of Interferon and five nucleoside/nucleotide analogs (NRTIs) licensed for the treatment of chronic HBV infection. Alpha IFN was licensed in 1992 and pegylated alpha IFN 2a was licensed in 2005. The five NRTIs are 3TC (1998), Adefovir (ADV; 2002), Entecavir (ETV; 2005), Telbivudine (LdT; 2006), and TDF (2008). FTC, which is structurally similar to 3TC, is also active against HBV and is frequently used for HBV treatment because it is co-formulated with TDF to treat HIV. 3TC, FTC, and LdT are L-nucleoside analogs; ADV and TDF are acylic nucleotide analogs; and ETV is a deoxyguanosine analog.

3TC, FTC, ADV, and TDF were each originally identified as antiretroviral drugs used for HIV-1. ETV, which was originally reported to be inactive against HIV-1 *in vitro*, was subsequently shown to reduce plasma HIV-1 RNA levels and to select for the RT mutation M184V in HIV-1 co-infected patients [100].

### 6.1. Interferon (IFN)

Although NRTIs are used more commonly than α-IFN, pegylated α-IFN is an important option for HBV treatment because unlike the NRTIs, a 24 to 48 week course of therapy is associated with an increased likelihood of sustained virologic response and HBsAb^+^/HBsAg^−^ seroconversion. Recent pilot studies have also suggested that a combination of NRTIs plus pegylated α-IFN may induce higher rates of sustained response and HBsAb^+^/HBsAg^−^ seroconversion than pegylated α-IFN alone [181–184].

### 6.2. Nucleoside/Nucleotide RT Inhibitors (NRTIs)

The three-dimensional structure of HBV RT has not been solved because it has been difficult to obtain sufficient amounts of highly purified active protein. However, homology modeling with other polymerases, including HIV-1 RT, has shown that HBV RT contains regions similar to the fingers, palm, and thumb of HIV-1 and seven sub-domains that are conserved among many published polymerase enzyme sequences [185,186]. In 2001, a standardized numbering system for mutations was established for the RT part of the HBV *pol* gene [187].

3TC resistance during 3TC monotherapy develops in 15% to 30% of individuals treated for one year, 40% to 50% treated for three years, and 70% treated for five years [188–190]. High-level (>1,000 fold) 3TC resistance is caused by the mutations M204V/I, which are in the YMDD motif adjacent to two of the RT enzyme’s catalytic aspartates [191,192] and likely sterically inhibits HBV RT binding to 3TC [185]. M204 mutations are also frequently accompanied by compensatory mutations, particularly L180M and, less commonly, V173L and/or L80V/I [192–195] (Table 3). M204 mutations are also selected by LdT, albeit at a slower rate than 3TC: 11% *versus* 26% after two years of monotherapy [196].

Although both HIV-1 and HBV develop 3TC resistance by the substitution of an I or V for an M in their RT’s YMDD motif, the slower development of HBV resistance and HBV’s frequent requirement for compensatory mutations in addition to M204V/I contrasts with the rapid development of 3TC resistance by M184V/I alone in HIV-1-infection.

High-level ETV resistance requires M204V/I + L180M and two or three of the following additional mutations I169T, T184S/A/G, S202G/I, or M250V [197–201]. Virological failure and ETV resistance are exceedingly uncommon when ETV is used to treat NRTI-naïve patients [200]. Although ETV retains considerable antiviral activity against 3TC-resistant variants [199,202], the risk of virological failure and high-level ETV resistance is considerable in 3TC-resistant patients.

ADV resistance emerges more slowly than 3TC resistance. It occurs in about 10% and 30% of individuals receiving ADV monotherapy for two and five years, respectively [189,203–205]. N236T and A181V/T, mutations close to the HBV active site, reduce ADV susceptibility by about 3 to 10-fold [204,206–209]. Although these reductions in susceptibility are much lower than the level of 3TC resistance conferred by M204V/I, they are associated with virologic breakthrough [206,207]. N236T causes partial cross-resistance to TDF but not to 3TC, LdT, or ETV [210].

ADV and TDF retain complete antiviral activity *in vitro* against viruses with the 3TC-resistance mutations: M204V/I, L180M, V173L, and L80I/V [208,211–213]. Indeed, these mutations may increase HBV susceptibility to ADV and TDF [214–217]. TDF has a high genetic barrier to resistance and the emergence of virological failure and TDF resistance is exceedingly uncommon when TDF is used to treat NRTI-naïve patients.

The HBV RT mutations A181V/T are unique in that they confer resistance to both L-nucleosides and acyclic nucleoside phosphonates and have emerged in individuals receiving ADV and, less commonly, 3TC [208,218–221]. A181T is of particular interest because it causes a stop codon in the reading frame coding for the surface protein, potentially allowing for ongoing hepatocellular replication without accompanying viral load rebound [222]. N236T, and to a lesser extent, A181V/T confer partial cross-resistance to TDF [223–225] and ETV is recommended for patients with these mutations [216].

## 7. HCV Drug Resistance

Although the combination of Peginterferon-α and Ribavirin is currently the only licensed treatment for HCV [106], many HCV-specific inhibitors are in advanced clinical development. Two PIs, telaprevir (TVR, formerly VX-950) and boceprevir (BVR, formerly SCH-503034) are in Phase III trials and at least 20 additional compounds—PIs, nucleoside inhibitors (NIs), nonnucleoside inhibitors (NNIs), an NS5A inhibitor, and a cyclophilin inhibitor—are in Phase II trials [232–235]. Improved *in vitro* systems that support intra-cellular replication have been essential to identifying HCV inhibitors and the genetic mechanisms of antiviral drug resistance [236–239].

### 7.1. Interferon (IFN) and Ribavirin

Pegylated α-IFN plus Ribavirin for six to 12 months is the standard treatment for HCV. Viral factors as well as host factors influence the response to IFN therapy. First, HCV genotype 2 and 3 viruses are significantly more likely than genotype 1 viruses to respond to IFN (sustained virologic response rates are about 70% for types 2 and 3 *versus* 45% for type 1) [105,106,240,241]. Second, therapy is more successful in acutely infected persons possibly because they harbor less-complex mixtures of quasispecies than do chronically infected persons [242,243]. Indeed, the likelihood of response to IFN is usually inversely proportional to the complexity of its quasispecies [244–248]. Third, specific mutational patterns in a 40-amino-acid region of genotype 1b NS5A have been associated with IFN responsiveness in several studies [249–251]. However, no specific mutations have been shown to be selected by or cause resistance to either IFN or Ribavirin [252–254].

A dose-response relationship exists between the nucleoside analog ribarivin and the likelihood of virologic suppression. Ribavirin interferes with dNTP metabolism by inhibiting cellular inosine monophosphate dehydrogenase [255,256] but may also directly inhibit HCV RNA polymerase [257], increase HCV mutagenesis [258,259], or modulate the HCV T cell immune response [256].

### 7.2. Protease Inhibitors (PIs)

The NS3 serine protease comprises the 189 N-terminal amino acids of NS3. NS3 forms a heterodimer with the 54-amino-acid NS4A cofactor. The HCV protease cleaves four sites in the HCV polypeptide precursor to generate the N termini of NS4A, NS4B, NS5A, and NS5B. Typical of other members of the trypsin family of serine proteases, NS3/4A contains a catalytic triad composed by H57, D81, and S139. Multiple three-dimensional structures of NS3/4A with and without inhibitors have been determined [260].

**Figure 2:**
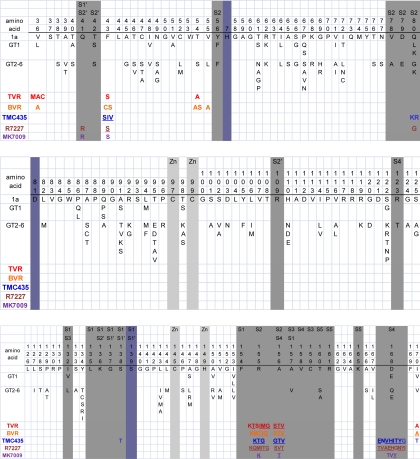
HCV NS3 Protease Variability and Protease Inhibitor (PI) Resistance Mutations. Alignment of NS3 residues 36 to 170 showing: (i) The consensus genotype 1a sequence and common variants in genotype 1 (GT1) and genotypes 2 to 6 (GT2–6) according to [277]; (ii) The active site residues are shaded blue-grey; (iii) The substrate binding site positions are shaded grey. The subsite numbering was derived from the following references: [278–282]; (iv) Mutations selected by specific PIs and/or associated with decreased PI susceptibility are indicated beneath the alignment. Underlined positions have been reported to decrease susceptibility >10-fold. PI abbreviations: Telaprevir (TVR), boceprevir (BVR), danoprevir (R7227), and vaniprevir (MK-7009). TMC435 does not have a generic name.

The HCV protease is a challenging drug target because it has a shallow substrate-binding pocket that normally binds a long peptide substrate with which it forms multiple weak interactions [261]. Sequence analysis of individual cleavage sites indicates that the intermolecular consensus sequence is relatively non-conserved: D/E-X-X-X-X-C/T↓A/S-X-X-X where X indicates multiple allowable residues [261,262]. Most HCV PI-resistance mutations occur within or near the substrate binding cleft particularly in the P1 to P4 binding pockets (S1 to S4 subsites; Figure 2). PI-resistance mutations selected *in vitro* have generally been predictive of those mutations selected *in vivo* [263].

The PIs, TVR and BVR are linear peptidomimetics that bind covalently but reversibly to the active site serine. When combined with α-IFN plus ribavirin, TVR and BVR increase the frequency of sustained virologic response by about 25% compared with placebo [264–267].

PI resistance occurs commonly in those patients who do not achieve a sustained virologic response. The protease mutations associated with resistance to TVR and BVR are nearly completely overlapping. They include V36A/M/L/C, T54A/S, R155K/T, A156S/V/T, and V170A (Table 4) [233,263,268–270]. The emergence of two PI-resistance mutations is generally sufficient for high-level resistance and virological failure. Several macrocyclic PIs including TMC435 [271], vaniprevir (formerly MK-7009) [272], and danoprevir (formerly ITMN-191 and R7227) [273] that do not covalently bind the active site serine are also in Phase II clinical trials. *In vitro* selection and drug S1’susceptibility studies show that Q41R, F43S, R155K/T, A156S/V/T, and D168A/E/H/T/V/Y are the most important mutations for these inhibitors.

Although the mutations associated with HCV PI resistance are for the most part conserved in genotype 1 viruses, sporadic mutations at these positions have been reported both as majority variants detectable by standard sequencing and as minority variants detected by deep sequencing methods [274,275]. In two studies of HCV protease sequences from more than 1,000 individuals with genotype I viruses, R155K was found in 0.7% of patients and V36M, T54A, D168E, and V170A were found in about 0.5% of patients [274,275]. Specific genotype-associated variants occur at accessory PI-resistance positions [276] and current PIs may have considerably decreased activity against viruses belonging to non-genotype 1 viruses [233].

### 7.3. Nucleoside (NI) and Non-nucleoside (NNI) Inhibitors

HCV RdRp is encoded by the 530 N-terminal amino acids of the NS5B gene. A C-terminal extension of NS5B anchors the catalytic domain to the endoplasmic reticulum as part of a larger viral replication complex that includes the NS3 RNA helicase. HCV RdRp, like other polymerases, contains palm, thumb, and finger subdomains that enclose the RNA template groove and a GDD catalytic triad [260]. HCV RdRp inhibitors include chain-terminating nucleoside analogs (NIs) and non-nucleoside analogs (NNIs) that target NS5B allosterically. Most HCV NIs differ from HIV-1 and HBV NRTIs in that chain termination is caused by steric hindrance rather than the absence of the 3’-hydroxyl group [68].

In contrast to the HCV PIs, NIs appear to be active against each of the HCV genotypes. The genetic barrier to NI resistance is higher than for the PIs and NNIs with prolonged *in vitro* passage required for the emergence of resistance [283]. Furthermore, the mutations associated with NI resistance generally reduce viral fitness to a greater extent than the NNIs and PIs [284,285]. Two non-cross-resistant mutational patterns associated with NI resistance have been described (Table 4): the active site mutation S282T has been selected *in vitro* by 2’-C-methyl modified nucleoside analogs including valopcitabine (NM203, an oral prodrug of the nucleoside analog 2’-C-methylcytidine), R7128 (a pro-drug of PSI-6130), and MK-0608 (2’-C-methyl-7-deaza-adenosine) [284,286]. S282T appears to sterically inhibit NIs containing 2-methyl-substituded nucleoside analogs [287]. S96T ± N142T, which are far from the active site, are selected *in vitro* by R1626 (a prodrug of R1479, 4’-azidocytidine) [288].

In a Phase II trial, the combination of R1626, Peginterferon alfa-2a, and Ribavirin led to complete virologic suppression by week four in nearly 75% of patients without selecting for S96T ± N142T, the R1626-resistance mutations [289]. In another dose-finding study of 32 patients receiving RG7128 monotherapy for two weeks and 85 patients receiving RG7128 for one month, the vast majority of patients experienced continuous virus load decline proportional to the RG7128 dose without evidence for the emergence of S282T, the R7128-resistance mutation. Similarly, in a dose-ranging study of MK-0608 administered intravenously and orally to chimpanzees for 37 days, plasma HCV levels displayed marked reductions in plasma HCV levels with the development of minority populations of S282T in two chimpanzees.

Although R1626 and valopcitabine have been withdrawn because of toxicity [232], the limited clinical experience with R1626 and the ongoing studies with RG7128 and MK-0608 demonstrates the potential of the HCV RdRp NI class. The absence of significant *in vivo* resistance to HCV NIs contrasts with the frequent resistance to the NRTIs used in the treatment of HIV and HBV. In a recent two-week clinical trial of the RG7128 in combination with the PI danoprevir (INFORM-1), subjects experienced a 3.7 to 5.2 log_10_ decrease in HCV IU/mL. Not only did RG7128 mutations fail to emerge, the combination also appeared to prevent the emergence of resistance to the PI danoprevir over the two week trial period [290].

Investigational non-nucleoside inhibitors (NNIs) targeting four allosteric binding sites are in early clinical development [232,291–293]. Two of these sites are in the thumb subdomain and two are in the palm subdomain. Mutations associated with resistance to each of the four allosteric sites have been selected *in vitro* and/or *in vivo* [233] (Table 4). Cross-resistance between NNIs and NIs has not been described. However, NNIs have uniformly displayed a low genetic barrier to resistance [294–296] and the activity of NNIs has often been variable even within the same genotype [295,297]. Moreover, several NNI- resistance mutations have been identified in previously untreated individuals either as dominant variants detected by standard sequencing or as minor variants detected by more sensitive methods [275,297,298].

### 7.4. NS5A Inhibitors

NS5A is a 447 amino acid membrane-associated phosphoprotein that is an essential part of the HCV replicase complex and an antagonist of endogenous IFN. The structure of the N-terminal domain of NS5A has been crystallized but how this domain and the complete protein functions are not known [299,300]. BMS-790052 was identified by a high throughput screening approach for targeting non-enzymatic HCV targets. It has an EC50 of below 10 picomoles in genotype 1a and 1b replicons. It decreased HCV plasma RNA levels about 1,000 fold within 24 hours in a randomized, double-blind, single ascending dose study [235].

The specificity of BMS-790052 for NS5A was demonstrated by the selection of mutations in the N-terminal domain that conferred high-level BMS-790052 resistance [235,301,302]. A combination of two BMS-790052 mutations is required to cause high-level BMS-790052 resistance [302].

### 7.5. Cyclophilin Inhibitors

Cyclophilin A is an important cellular cofactor for HCV replication. Although the role of cyclophilin A in HCV replication is not known, it appears to a binding partner of NS5A and possibly other HCV proteins [303]. Debio 025 is a non-immunosuppressive cyclosporine analog that potently inhibits the interaction of cyclophilin A and HCV *in vitro* [303] and *in vivo* [304]. The genetic barrier to Debio 025 resistance is high [305] and may require the selection of cyclophilin A-independent NS5A variants [303]. Drugs that block the interaction of cyclophilin A with HCV are somewhat analogous to HIV CCR5 inhibitors in that the primary target of therapy is a host protein.

## 8. Conclusions

Nearly 25 drugs belonging to six drug classes have been licensed for treating HIV-1. In previously untreated individuals infected with drug susceptible HIV-1, combinations of three drugs from two drug classes leads to prolonged virus suppression. However, because HIV cannot be eradicated from its proviral DNA reservoir lifelong therapy is necessary. Prolonged therapy carries the risk that periods of nonadherence will lead to the selection of drug-resistance variants. This risk is particularly high in low-income countries where interruptions in drug supply occur, the regimens used have lower genetic barriers to resistance than those used in high-income countries, and laboratory monitoring is less intensive. Continued research is therefore required to develop fixed-dose drug combinations with high genetic barriers to resistance that can be administered safely for long periods of time.

Although the fewest therapeutic options are available for treating HBV, it is the only virus in this review for which monotherapy—with either ETV or TDF—is capable of fully suppressing virus replication for many years in previously untreated persons. HBV has generally not been considered eradicatable because of its cccDNA form. However, eradication will be attempted by studies that combine pegylated IFN-α and other investigational IFN formulations with the two most potent NRTIs—ETV and TDF.

The first two small molecule HCV inhibitors may be licensed in 2011 and many more are likely to follow in the next decade. The introduction of new inhibitors will increase the frequency of virological cures and reduce HCV morbidity and mortality. It will also lead to widespread acquired drug resistance among the patients who do not achieve sustained virologic response. However, as the number of licensed new non-cross-resistant inhibitors increases, virological failure will decrease in frequency, salvage regimens will be available for patients with resistance to the first generations of small molecule inhibitors, and IFN-sparing regimens will be increasingly used.

## Figures and Tables

**Table 1:** Human Immunodeficiency Virus (HIV), Hepatitis B Virus (HBV), and Hepatitis C Virus (HCV): Replication Characteristics and Antiviral Treatment.

**Virus**	**Genomic Classification**	**Intracellular Reservoir**	Mutation Rate*	**Plasma Levels†**	**Recombination**	**Antiviral Drug Classes§**
HIV	Retrovirus	Proviral DNA	10^−5^	10^3^ − 10^6^	Major contribution to virus evolution in individuals and populations	Nucleoside RT inhibitors Nonnucleoside RT inhibitors Protease inhibitors Integrase inhibitors Fusion inhibitors CCR5 inhibitors
HBV	DS DNA virus with obligate RNA intermediate	Nuclear covalently-closed circular DNA (cccDNA)	10^−5^	10^5^ − 10^9^	Possible contribution to virus evolution within individuals	Interferon Nucleoside RT inhibitors
HCV	Positive single-stranded RNA virus	None	10^−4^ − 10^−5^	10^4^ − 10^7^	Possible contribution to virus evolution within individuals	Interferon + Ribavirin Protease inhibitors Nucleoside inhibitors Nonnucleoside inhibitors NS5A inhibitors Cyclophilin inhibitors

* Mutation rates during a single round of replication have been estimated experimentally for HIV-1. For HBV and HCV these rates have been estimated from mathematical models and comparisons with other viruses.

† RNA copies per mL for HIV-1 and DNA copies per mL for HBV. Range encompasses the majority of untreated individuals with ongoing replication.

§ HCV protease inhibitors are in Phase III clinical trials. HCV nucleoside, nonnucleoside, NS5A, and cyclophilin inhibitors are in Phase II clinical trials.

**Table 2:** Mechanisms of Resistance to Human Immunodeficiency Virus Type 1 (HIV-1) Inhibitors.

**Drug Class**	**Mechanism of Resistance**	**Mutations**	**Drug Resistance Mutations**
Nucleoside/Nucleotide RT inhibitors (NRTIs): Abacavir (ABC) Didanosine (ddI) Emtricitabine (FTC) Lamivudine (3TC) Stavudine (d4T) Zidovudine (AZT) Tenofovir (TDF)	RT mutations that enhance discrimination between NRTIs and natural nucleosides	K65R, L74V, Y115F, Q151M, M184V	K65R causes high-level resistance to ddI, ABC, and TDF, intermediate resistance to 3TC and FTC, low-level resistance to d4T, and increased susceptibility to AZT. L74V decreases susceptibility to ddI and ABC. Y115F decreases susceptibility to ABC and TDF. Q151M causes high-level resistance to AZT, d4T, ddI, and ABC, and intermediate resistance to TDF, 3TC, and FTC. M184V causes high-level resistance to 3TC and FTC and low-level resistance to ABC and ddI. Reviewed in [177,178].
	RT mutations that promote ATP-dependent hydrolytic removal of chain-terminating nucleotide monophosphates(also known as thymidine analog mutationsor TAMs).	M41L, D67N, K70R, L210W, T215F/Y, K219Q/E T69S_SS	M41L, D67N, K70R, L210W, T215FY, and K219QE develop in viruses from patients receiving AZTand d4T. The accumulation of several TAMs causescross-resistance to each of the other NRTIs except 3TC and FTC. T69S_SS is an uncommon amino acid insertion that confers resistance to each of the NRTIs when it occurs in combination with multiple TAMs. Reviewed in [177,178].
Non-nucleoside RT inhibitors (NNRTIs): Efavirenz (EFV) Etravirine (ETR) Nevirapine (NVP)	Mutations in the HIV-1 RT NNRTI-binding pocket	L100I, K101E/P, K103N, V106A/M Y181C/I/V, Y188L G190A/S, M230L	These mutations cause high-level resistance to NVP and intermediate or high-level resistance to EFV. With the exception of K103N, V106A/M, and Y188L, each mutation is also associated with decreased ETR susceptibility. Reviewed in [136,141,177].
Protease inhibitors (PIs): Atazanavir (ATV) Darunavir (DRV) Fosamprenavir (FPV) Indinavir (IDV) Lopinavir/r (LPV/r) Nelfinavir (NFV) Saquinavir (SQV) Tipranavir (TPV)	Protease mutations interfere with inhibitor binding or compensate for the decreased replication associated with other mutations.	D30N, V32I, V47V/A, G48V, I50V/L, I54M/L/V/A/T, L76V, V82A/T/F/S/L, I84V/A, N88S, L90M	Positions 30, 32, 47, 48, 50, 82, and 84 are in the substrate cleft. Position 54 is in the flap and directly interacts with PIs as they enter the substrate cleft. The mutations at positions 76, 88, and 90 influence the shape of the substrate cleft indirectly. Reviewed in [142,177].
	These mutations are primarily compensatory	L10I/V/F, L24I, L33F, M46I/L F53L, A71V/T/I/L, Q58E, G73S/T/C/A, T74P, N83D, N88D, L89V	L10I/V, L33F, M46I/L, and A71V/T are minimally polymorphic occurring in 0.5% to 5% of viruses from untreated persons depending on the subtype. Reviewed in [142,177].
Integrase inhibitors (INIs): Raltegravir (RAL) In Phase III trials: Elvitegravir (EVG) S/GSK1349572 (572)	Mutations in residues surrounding the IN active site.	Q148H/R/K ± G140SA, N155H ± E92Q, Y143C/R, T66I/A/K, S147G	Q148H/R/K ± G140SA cause high-level RAL and EVG resistance and intermediate 572 resistance. N155H + E92Q causes high-level RAL and EVG resistance. Y143C/R + T97A causeshigh-level RAL resistance. T66I and S147G are selected in patients receiving EVG and decrease EVG susceptibility but do not appear to cause RAL cross-resistance. Reviewed in [179].
Fusion inhibitors: Enfuvirtide (ENF)	Mutations in the first heptad repeat region (HR1) of the gp41 transmembrane protein interfere with the association of HR1 and HR2 required for virus cell fusion.	G36D/E/V/S, I37V, V38E/A/M/G, Q48H, N42T, N43D/K/S, L44M, L45M	G36D/E, V38E/A, Q40H, and N43D each reduce ENF susceptibility >10-fold [168,169]. Two mutations are usually sufficient to cause high-level ENF resistance.
CCR5 inhibitors: Maraviroc (MVC)	Virological failure and resistance is usually caused by expansion of pre-existing CXCR4-tropic variants that were not detected at the start of therapy. *In vitro*, and occasionally, *in vivo* resistance is caused by gp120 mutations that facilitate binding to an inhibitor bound CCR5 molecule.	Positively charged residues at positions 11 and 25 of the V3 loop of gp120 and many other combinations of mutations primarily but not exclusively within the V3 loop are associated with CXCR4 tropism [180]. No consistent pattern of gp120 mutations has been identified to be associated with virus binding to an inhibitor-bound CCR5 receptor [174–176].	

**Table 3:** Mechanisms of Resistance to Hepatitis B Virus (HBV) Inhibitors.

**Antiviral Agents**	**Mechanism of resistance**	**Mutations**	**Drug Resistance**
Interferon	Unknown	Unknown	Unknown
Lamivudine (3TC) Telbivudine (LdT) Emtricitabine (FTC)^*^ Entecavir (ETV) Adefovir (ADV) Tenofovir (TDF)	RT mutations that interfere with nucleotide triphosphate binding. Whether any of these mutations also facilitate primer unblocking is not known.	M204V/I ± L180M ± L80I, V173L	M204V/I ± L180M and less commonly L80I and V173L emerge during 3TC treatment and confer cross-resistance to LdT and FTC; and partial cross-resistance to ETV. M204V/I also emerge during LdT therapy. Reviewed in [215,223,226].
		N236T	Selected by ADV and causes partial cross-resistance to TDF. Reviewed in [215,223,226].
		A181V/T	Selected by ADV and less commonly 3TC. May causes partial cross-resistance to TDF but not ETV. Reviewed in [215,223,226].
		I169T, T184S/A/G, S202G/I, M250V	Selected by ETV particularly in viruses with pre-existing 3TC-resistance mutations. Reviewed in [215,223,226].

* FTC is not licensed for HBV treatment. However, it is frequently used in combination with Tenofovir for salvage therapy because there is a co-formulated version of TDF and FTC (Truvada) licensed for the treatment of HIV-1. Several mutations are not shown because they are either extremely rare (e.g., M204S, A181S) or because their association with resistance is controversial: e.g., A233V for Adefovir [227–229], and A194T for Tenofovir [230–231].

**Table 4:** Mechanisms of resistance to Hepatitis C Virus (HCV) inhibitors.

**Antiviral Agents**		**Mechanism of resistance**	**Mutations**	**Drug Resistance**
Interferon-α		Unknown		Genotype 1 isolates respond less well than genotype 2 or 3 viruses but the molecular basis is not known.
Ribavirin		Unknown		Unknown
PIs: Telaprevir (TVR) Boceprevir (BVR) TMC435 Danoprevir Vaniprevir		Mutations within or near the protease substrate cleft	V36A/M/C, Q41R, F43S/I/V, T54A/S, Q80K/R, R155K/T, A156S/V/T, D168A/E/I/N/T/V/Y, V170A/T	R155K/T and A156S/V/T decrease susceptibility to all PIs. V36A/M/C and T54A/S decrease susceptibility to the linear peptidomimetics TVR and BVR. V170A is selected by BVR but may cause cross-resistance to TVR. Q41R, F43S/I/V, and D168 mutations decrease susceptibility to TMC435, danoprevir, and vaniprevir. Q80K, a common polymorphism in genotype 1a, and V170T decrease TMC435 susceptibility about 5-fold. [233,263,268,271,272]
NIs: NM203 (withdrawn) R1626 (withdrawn) R7128 MK-0608		Steric hindrance of nucleoside analog incorporation (S282T)	S282T	S282T in combination with compensatory mutations has been selected *in vitro*by 2’-C-methyl modified NIs including valopcitabine (NM203, an oral prodrug of the nucleoside analog 2’-C-methylcytidine) and R7128 (a pro-drug of PSI-6130).
			S96T ± N142T	S96T ± N142T are selected *in vitro* by R1626 (a prodrug of R1479, 4’-azidocytidine). These mutations are far from the HCV polymerase active site. R1626 has been withdrawn from clinical development [288].
NNIs		Decreased binding to NNI I pocket (upper thumb)	P495S/A/L, P496S/A, V499A	NNI site 1 is about 30Å from the active site [306]. A series of benzimidazole 5-carboxamide compounds bind to this site [292,307,308]. GS9190, BI207127, and MK3281 are site 1 NNIs in clinical development [68,233]. Mutations at positions 495, 496, and 497 reduce susceptibility to site 1 NNIs [68,233].
		Decreased binding to NNI site 2 (base of thumb)	L419V/M, M423T/V/I, I482L/V/T, V494A/I	NNI site 2 is a shallow hydrophobic pocket at the base of the thumb close to NNI site 1 and ∼35Å from the active site. Compounds that bind to this site such as filibuvir, VC-759, and VCH-796 have selected the mutations L419M, M423T/V/I, I482L, and V194A [68,233,294,309].
		Decreased binding to NNI site 3 (inner thumb / palm)	H95R, M414T, C451R, G554D, G558R, D559G	Benzothiadizine compounds that bind to this site have selected for M414T, C451R and G558R [310]. ANA598 is a site 3 NNI in Phase II trials [233]. Mutations associated with this drug have include M414T, G554D, and D559G [233]. M414T, which is polymorphic in genotypic 1 viruses, may play a role in resistance to both site 3 and site 4 NNIs.
		Decreased binding to NNI site 4 (palm)	C316N/Y, S365T, L392F, M414T, Y448H	C316Y is selected rapidly *in vitro* by HCV-796 [311], a site 4 NNI that is no longer in clinical development. Other mutations that have been selected by HCV-796 include C316N, S365T/A, L392F, and M414T [233,311]. ABT-333 is a site 4 NNI that has selected for C316N/Y and Y448H [233].
NS5A inhibitors: BMS-790052		Unknown	M28T, Q30E/H/R, L31M/F/V, P32L, Y93C/H/N	In selection experiments with BMS-790052, M28T, Q30E/H/R, L31M/V, P32L, and Y93C/H/N have been selected in a genotype 1a replicon. L31F/V, P32L, and Y93H/N have been selected *in vitro* in a genotype 1b replicon. Two mutations are usually required for high-level resistance [302].
Cyclophilin inhibitors: Debio 025	Unknown		Unknown	

* All compounds other than IFN and Ribavirin are in clinical development.
